# Enhancement of the antioxidant and skin permeation properties of eugenol by the esterification of eugenol to new derivatives

**DOI:** 10.1186/s13568-020-01122-3

**Published:** 2020-10-19

**Authors:** Edyta Makuch, Anna Nowak, Andrzej Günther, Robert Pełech, Łukasz Kucharski, Wiktoria Duchnik, Adam Klimowicz

**Affiliations:** 1 Department of Chemical Organic Technology and Polymeric Materials, Faculty of Chemical Technology and Engineering, West Pomeranian University of Technology, Szczecin, Pulaskiego 10, 70–322 Szczecin, Poland; 2 Department of Cosmetic and Pharmaceutical Chemistry, Pomeranian Medical University in Szczecin, Powstańców Wielkopolskich Ave. 72, 70–111 Szczecin, Poland

**Keywords:** Eugenol, New esters of eugenol, log P, Skin penetration, Franz cell, Antioxidant activity

## Abstract

The aim of the study was to determine the antioxidant activity and assess the lipophilicity and skin penetration of eugenyl chloroacetate (EChA), eugenyl dichloroacetate (EDChA), and eugenyl trichloroacetate (ETChA). Identification of the obtained products was based on gas chromatography (GC), infrared spectroscopy (FTIR/ATR), gas chromatography coupled with mass spectrometry (GC-MS), and the analysis of ^13^C-NMR and ^1^H-NMR spectra. The antioxidative capacity of the derivatives obtained was determined by the DPPH free radical reduction method, while the octanol/water partition coefficient (*shake-flask* method) was tested to determine the lipophilicity of these compounds. In the next stage of testing EDChA and ETChA–(compounds characterized by the highest degree of free radical scavenging), the penetration of DPPH through pig skin and its accumulation in the skin were evaluated. For comparison, penetration studies of eugenol alone as well as dichloroacetic acid (DChAA) and trichloroacetic acid (TChAA) were also carried out. The antioxidant activity (DPPH, ABTS, and Folin–Ciocalteu methods) of the fluid that penetrated through pig skin was also evaluated. The in vitro pig skin penetration study showed that eugenol derivatives are particularly relevant for topical application. The obtained derivatives were characterized by a high level of antioxidant activity estimated after 24 h of conducting the experiment, which indicates long-term protection against reactive oxygen species (ROS) in the deeper layers of the skin.
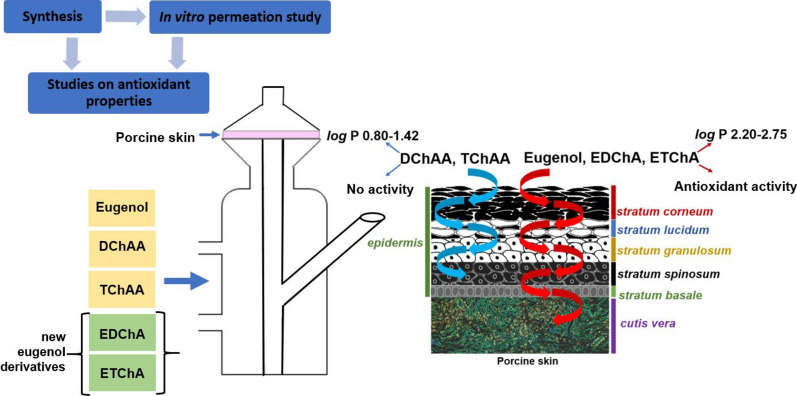

## Key points

- New esters of eugenol show potent antioxidant activity in vitro.
- There are new eugenol derivatives to penetrate easily through biological membranes.
- Presented esters can provide exogenous and endogenous action against free radicals.

## Introduction

Reactive oxygen species (ROS) are important factors in the ageing process that are formed during incomplete reduction of oxygen molecules in the respiratory chain. When there is an imbalance between reactive oxygen species and the body’s antioxidant potential, oxidative stress occurs. It is widely recognized that reactive oxygen species (ROS) contribute to the aging of the skin, which is the external barrier of our body. However, many tissues inside our body are also subjected to ROS. In fact, the human body is exposed to both endogenous and exogenous ROS effects. These compounds, which cause oxidative stress, are responsible for oxidative modifications of polyunsaturated fatty acids and nucleic acids (as a consequence, this leads to structural changes in cell membranes as well as to DNA damage). Antioxidants are compounds characterized by the ability to deactivate free radicals responsible for the ageing process of the body (Dhale et al. 2007; Michalak et al. 2014; Igielska-Kalwat et al. 2015; Agati et al. 2012; Zhao 2015; Suvarnakuta et al. 2011; Li and Seeram 2010; Janiuk et al. 2013). To assess their antioxidant properties, in vitro methods are mainly used, and these are based on two types of reactions: the HAT (hydrogen atom transfer) technique and the SET (single electron transfer) method. The first method is related to the transfer of a hydrogen atom derived from an antioxidant, while the second method involves the transfer of a single electron from an antioxidant molecule (Zheng 2017; Makuch et al. 2019). The most commonly used methods for assessing the antioxidant properties of compounds include the ferric ion reducing antioxidant parameter (FRAP) method, the method using the ABTS reagent (2,2’-azino-bis(3-ethylbenzothiazoline-6-sulfonic acid), determination of total polyphenol content using the Folin–Ciocalteu reagent, and the method for determining the antioxidant activity using the DPPH radical (2,2-diphenyl-1-picrylhydrazyl) (Gulcin 2011; Molyneux 2004; Li et al. 2011; Benzie and Strain 1996). In spectroscopic methods, the quantitative analysis of antioxidant capacity is based on the change in absorbance of the test solution (Gulcin 2011; Molyneux 2004; Li et al. 2011; Benzie et al. 1996).

The DPPH radical is a dark purple color and has a maximum absorbance at λ = 515–517 nm. The method of measuring the antioxidant capacity of compounds using the DPPH technique is based on a spectrophotometrically recorded change in the color of the ethanol solution of the DPPH radical. The reaction of the DPPH radical with an antioxidant occurs on one of the nitrogen atoms present in the DPPH molecule (on the so-called nitrogen bridge). When the DPPH radical is transferred to the hydrogen atom present at the antioxidant hydroxyl group, the dark violet color of the DPPH ethanol solution changes to light yellow or the solution completely discolors; thus, the absorbance of the tested solution decreases (Gulcin 2011; Molyneux 2004; Li et al. 2011).

Literature reports indicate that clove oil, eugenol, and esters obtained by esterifying eugenol with acetic anhydride or benzoic acid (eugenyl acetate and eugenyl benzoate) are characterized by the ability to deactivate free radicals (Horchani et al. 2010; Vanin et al. 2014; Sá et al. 2017). Besides, eugenol, eugenyl acetate, eugenyl propionate, eugenyl butyrate and eugenyl 4-ethyl, 4-fluoro, 4-chloro and 4-bromobenzoate are also characterized by antimicrobial activity (Rahim 2017; Chaibakhsh et al. 2012; Cichorek et al. 2013). Other literature reports indicate that eugenol and eugenyl acetate are also characterized by anti-tumour activity against the B16 melanoma cell line, as assessed by the method described by Arung et al.  (2007, 2011).

The results of our previous preliminary studies related to the assessment of antioxidant activity showed that products obtained by the esterification of eugenol with the appropriate carboxylic acid chloride compounds are characterized by their ability to react with the free radical DPPH (Makuch et al. 2019). The natural consequence of this trend is the synthesis and evaluation of antioxidant activity of the chloro-, dichloro-, and trichloroacetate derivatives. In addition to antioxidant activity, antioxidants should be characterized by an adequate level of penetration through the stratum corneum of the skin into its inner deeper layers. The indicator of the hydrophilicity and hydrophobicity of compounds is the octanol/water partition coefficient (log P). By increasing the lipophilicity of eugenol through its esterification, the penetration of obtained ester derivatives through cell membranes is facilitated (Malinowska et al. 2013). The stratum corneum is a physical, environmental, and microbiological barrier that protects the body against external factors. It is mainly made of lipid substances such as ceramides, cholesterol, fatty acids, cholesterol esters, and phospholipids present in the stratum corneum in small amounts. These substances can significantly inhibit the penetration of exogenous compounds (both therapeutic and cosmetic) into the deeper layers of the skin. Also, the nature of the outer layer means that lipophilic substances penetrate it much more easily than the hydrophilic compounds that accumulate in it (Jaworska et al. 2011). By giving active substances adequate lipophilicity, their penetration is increased while their accumulation is limited. This phenomenon is particularly important in the case of active substances such as added antioxidants that are characterized by high antioxidant activity in both the stratum corneum of the skin and in its deeper layers (IIntarakumhaeng and Li 2014; Zhang et al. 2016). Therefore, the use of eugenol ester derivatives as active substances with high antioxidant activity and higher lipophilicity (compared to eugenol and the acids from which these derivatives were obtained) can be an alternative for obtaining cosmetic preparations characterized by long-lasting protection against free radicals (ROS).

As part of the research, eugenyl chloro-, dichloro-, and trichloroacetate were obtained by reacting eugenol with chloro-, dichloro-, and trichloroacetic acid chlorides. Esters were synthesized as described in our previous publication (Makuch et al. 2019). The selectivity of the conversion to EChA, EDChA, and ETChA as well as the conversion of eugenol were determined by the gas chromatography (GC) method, while the molar masses of the obtained products were confirmed based on the mass spectrum (GC-MS). The most important band associated with the presence of an ester group in the structure of the obtained products was identified by infrared spectroscopy. The unequivocal confirmation of the structure of new eugenol ester derivatives not yet described in the literature was carried out by NMR. The antioxidative activity and the octanol/water partition coefficient of eugenol and its esters were evaluated by the spectrophotometric method.

The penetration through pig skin using the Franz diffusion cell, accumulation of two derivatives (EDChA and ETChA) in the skin, eugenol content, as well as DChAA and TChAA contents were evaluated. Moreover, the antioxidant potential of solutions applied to the skin and obtained after 24 h of penetration through the skin as well as the activity of the skin after the test were assessed.

## Materials and methods

### Chemicals

The following compounds were used for the synthesis of eugenol esters: chloroacetic acid (Sigma-Aldrich, 99%), dichloroacetic acid (Sigma-Aldrich, ≥ 99%), trichloroacetic acid (Chempur, 99%), oxallyl chloride (Alfa Aesar, 98%), eugenol (Keten, p.a.), pyridine (AR Ubichem), dimethylformamide (DMF, Acros, 99%), chloroform (Chempur, p.a.), and deionized water.

The amounts of these compounds were selected to maintain the molar ratio of carboxylic acid:oxalyl chloride:eugenol:amines as 81:71:51:123. For the determination of antioxidant activity and to assess the lipophilicity of the esters obtained, 2,2-diphenyl-1-picrylhydrazyl (DPPH), 6-hydroxy-2,5,7,8-tetramethylchroman-2-carboxylic acid (trolox), 2,2′-azino-bis(3-ethylbenzothiazoline-6-sulfonic acid) (ABTS), and sodium lauryl sulfate were purchased from Sigma Aldrich (USA); Folin–Ciocalteu reagent, disodium phosphate (p.a.), and potassium dihydrogen phosphate (p.a.) were purchased from Merck, Darmstadt, Germany; ethanol (96% v/v), methanol (50% v/v, concentrated) (all of the analytical grade) sodium chloride, potassium chloride, and gallic acid (GA) were purchased from Chempur, Piekary Śląskie (Poland); and acetonitrile for HPLC was purchased from J.T. Baker, PBS.

### Synthesis and characterization of new esters

First, EChA, EDChA, and ETChA were obtained by the method described in our previous publication (Makuch et al. 2019). After synthesis, the obtained products were identified by mass spectrometry coupled with gas chromatography (GC-MS). Chromatographic analyses were performed with a TRACE GC series apparatus with a VOYAGER mass detector using a DB5 capillary column (30 m × 0.25 µm × 0.5 µm). The following separation parameters were used for the analysis: helium flow of 1.0 ml/min, sample chamber temperature of 240 °C, and detector voltage of 350 V. The thermostat temperature increased according to the following program: isothermal at 50 °C for 1 min, increase at a rate of 8 °C/min, isothermal at 260 °C for 5 min, and then cooled to 50 °C. The sample partition coefficient in the dispenser was 20, the volume of dispensed sample was 0.1 µl, and the ion mass range was 25–350 mV/z.

The degree of conversion of eugenol and the purity of the obtained products were determined by gas chromatography (GC). Chromatographic analyses were performed on a FOCUS apparatus (Thermo Electron) with a flame ionization detector (FID) and a RESTEK RTX-5 capillary column (0.53 mm × 30 m × 1.5 µm). Infrared spectroscopy (FTIR/ATR) was characterized by functional groups present in the structures of the products obtained. Analyses were performed on a THERMO NICOLED 380 apparatus using a measuring range of 4000–400 cm^−1^. The same method also used eugenol for comparative purposes.

The structures of the obtained esters (dissolved in deuterated chloroform (CDCl_3_)) were confirmed based on the analysis of nuclear magnetic resonance (NMR) spectroscopy spectra, which was performed with a Bruker DPX-400 spectrometer. The conditions for recording ^13^C-NMR spectra were as follows: 100.62 MHz, a spectrum width of 24 kHz, 65.5 K data points, a resolution of 1.46 Hz/point, a data acquisition time of 1.37 s, a repetition time of 1 s, a pulse width of 9.2 µs, and 1–8 scans. In addition, ^1^H-NMR spectra were recorded under the following conditions: 400.13 MHz, a 12 kHz spectrum width, 65.5 K data points, a 0.488 Hz/point resolution, a data acquisition time of 4.09 s, a repetition time of 1 s, a 7.8 µs pulse width, and 16–32 scans.

**Fig. 1:**
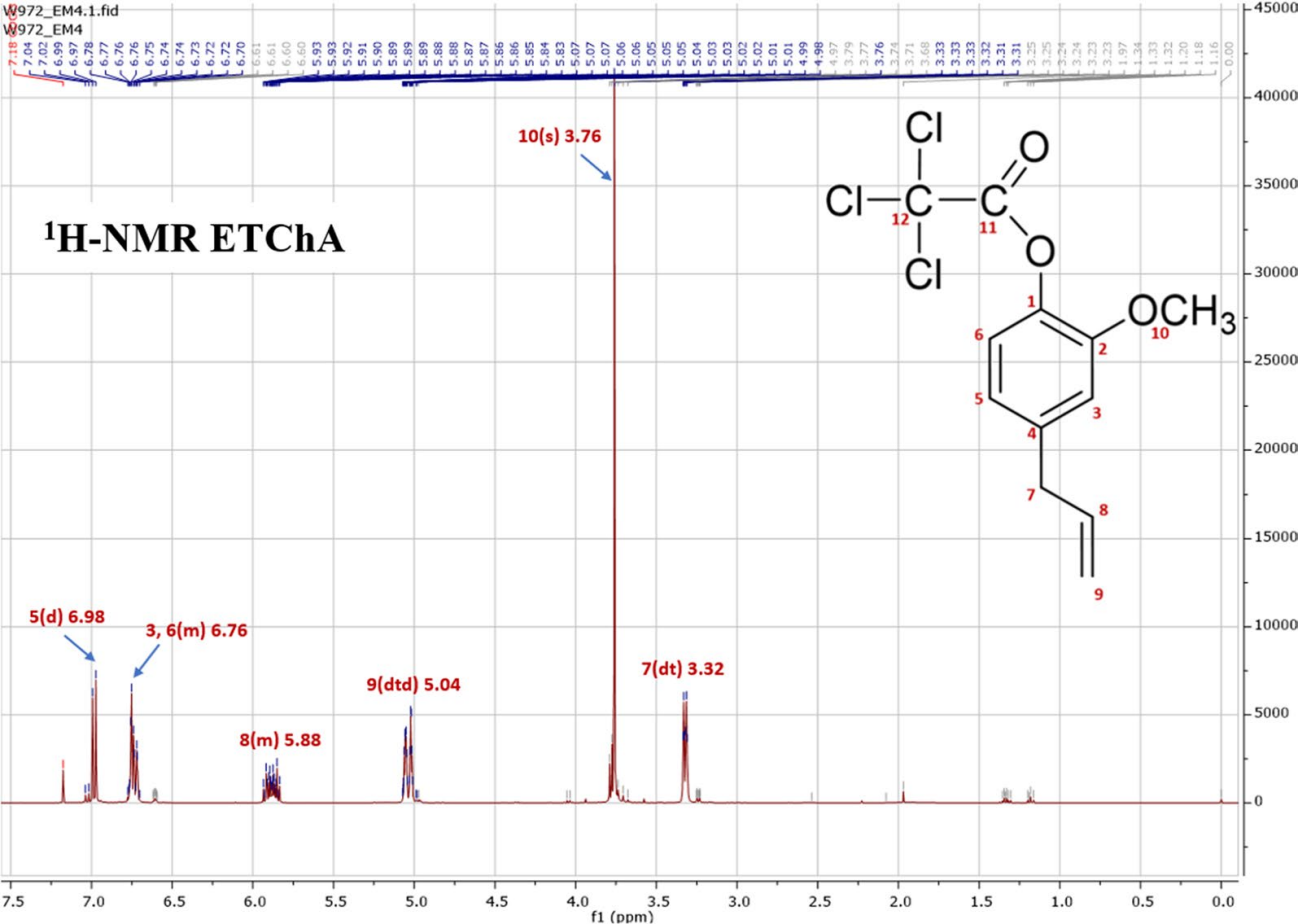
^1^H-NMR spectrum of ETChA

**Fig. 2:**
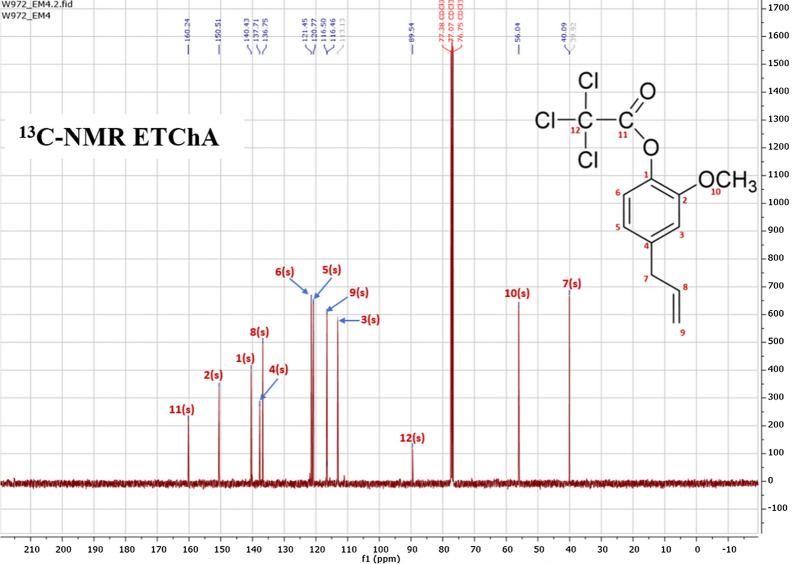
^13^C-NMR spectrum of ETChA

On the ^1^H-NMR spectrum, which is presented in Fig. 1, the presence of clear peaks indicating the presence of certain groups of protons in the structure of the tested ester was observed. The proton signals recorded as intense peaks corresponded to hydrogen atoms from groups belonging to hydrocarbon chains, indicating the presence of an aromatic system in the molecule of the compound tested. The analysis of the ^13^C-NMR spectrum presented in Fig. 2 supplemented the information obtained based on the interpretation of the ^1^H-NMR spectrum and additionally confirmed the structure of the obtained ester. The chemical shift values of carbon atoms and protons as well as the signals assigned to atoms ^13^C and ^1^H were as follows:

^13^C-NMR (101 MHz, CDCl_3_) δ 160.24, 150.51, 140.43, 137.71, 136.75, 121.45, 120.77, 116.50, 116.46, 89.54, 56.04, 40.09;

^1^H NMR (400 MHz, CDCl_3_) δ 6.98 (d, *J* = 8.0 Hz, 1H), 6.80–6.67 (m, 2H), 5.97–5.81 (m, 1H), 5.04 (dtd, *J* = 14.7, 3.4, 1.7 Hz, 2H), 3.76 (s, 3H), 3.32 (dt, *J* = 6.7, 1.5 Hz, 2H).

For the remaining esters, the chemical shift values of carbon atoms and protons and the signals assigned to atoms ^13^C and ^1^H were as follows:

^13^C-NMR (101 MHz, CDCl_3_) δ 165.62, 150.59, 139.66, 137.55, 136.91, 122.13, 120.72, 116.34, 112.82, 55.86, 40.71, 40.10 - (EChA);

^1^H NMR (400 MHz, CDCl_3_) δ 6.90 (d, *J* = 7.9 Hz, 1H), 6.80–6.65 (m, 2H), 5.88 (ddt, *J* = 17.0, 10.3, 6.8 Hz, 1H), 5.11–4.97 (m, 2H), 4.25 (s, 2H), 3.73 (s, 3H), 3.30 (dt, *J* = 6.8, 1.5 Hz, 2H) - (EChA);

^13^C-NMR (101 MHz, CDCl_3_) δ 162.84, 150.65, 140.24, 137.42, 136.91, 121.81, 120.86, 116.55, 113.12, 64.14, 56.08, 40.20 - (EDChA);

^1^H NMR (400 MHz, CDCl_3_) δ 7.02 (d, *J* = 8.0 Hz, 1H), 6.83–6.80 (m, 1H), 6.78 (q, *J* = 1.9 Hz, 1H), 6.19 (s, 1H), 5.96 (ddt, *J* = 9.4, 17.6 Hz, 1H), 5.11 (dt, 2H), 3.83 (s, 3H), 3.39 (dd, *J* = 1.6, 6.8 Hz, 2H) - (EDChA).

### Measurement of lipophilicity of eugenol and new esters

To determine the lipophilicity of eugenol and its ester derivatives, the values of the n-octanol/water partition coefficient (P) were examined. Determining the lipophilicity of a substance involves determining its partition coefficient between two immiscible liquids: n-octanol and water (which model the properties of cell structures well). The partition coefficient was expressed as the logarithm ratio of substance concentrations in both phases (Piwowarczyk et al. 2017):

$$\text{log} \,\text{P} = \text{log}\, \text{C}_{\text{n-octanol}} - \text{log} \,\text{C}_{\text{water}},$$

where P is the partition coefficient, C_n-octanol_ is the concentration of the compound in the octanol phase, and C_water_ is the concentration of the compound in the aqueous phase.

N-octanol was mixed with water in a 1:1 ratio containing the test compound at a concentration of 32.00–178.00 mg/100 ml solution. The mixture was then shaken on a shaker (TS-2 Orbital Shaker) for 24 h at a constant temperature of 25 °C, which was controlled by an immersion thermostat. Concentrations of substances in the analyzed samples were determined by spectrophotometry at the following wavelengths: 279 nm (in the case of eugenol), 272 nm (in the case of EChA), 277 nm (in the case of EDChA), and 280 nm (in the case of ETChA). For comparison purposes, log P values for DChAA and TChAA were also determined by potentiometric method. Furthermore, blanks were performed for each compound tested. For this purpose, n-octanol was mixed with water in a 1:1 ratio, and then the mixture was shaken for 24 h under constant temperature conditions, after which the aqueous layers were analyzed by a spectrophotometric method using appropriate wavelengths λ (Piwowarczyk et al. 2017).

Furthermore, compounds contained in aqueous solutions were identified by thin-layer chromatography (TLC). Based on the obtained chromatograms, the retention factor (R_f_) values for the corresponding ester and pure eugenol were determined. The absence of eugenol in aqueous solutions of the esters tested indicated a lack of the hydrolysis of these compounds (Waszczuk and Makuch et al. 2020).

### Measurement of the antioxidant capacity using DPPH, ABTS and Folin–Ciocalteu methods

Studies on the antioxidant activity of eugenol and its esters were carried out by free radical reduction (DPPH) (Brand-Williams et al. 1995; Nowak et al. 2017), ABTS (Nowak et al. 2017), and the Folin–Ciocalteu method (Nowak et al. 2019). The ABTS assay is based on the generation of a blue/green ABTS, which is applicable to both hydrophilic and lipophilic antioxidant systems; whereas DPPH assay uses a radical dissolved in organic media and is, therefore, applicable to hydrophobic systems (Floegel et al. 2011).

The analyses were performed on a Merck Spectroquant Pharo 300 apparatus at the following wavelengths λ: 517 nm (in the case of the DPPH method), 734 nm (in the case of the ABTS method), and 765 nm (in the case of the Folin–Ciocalteu method). Trolox was used as a reference substance in the DPPH and ABTS methods, while gallic acid was used as a standard to assess the polyphenol contents in the sample tested by the Folin–Ciocalteu method. The antioxidant activity results obtained by these methods are expressed in mmol/dm^3^.

The antioxidant activity of eugenol and its ester derivatives was measured as follows: 2850 µl of an ethanol solution with the DPPH radical was introduced into the tube, and its absorbance at λ = 517 nm was about 1.000 ± 0.020 with 150 µl of the ethanol solution containing the tested antioxidant. The tube was wrapped in aluminum foil and its contents were sealed with a stopper and then incubated for 10 min at room temperature. After this time, spectrophotometric measurements were carried out at the appropriate wavelength and in triplicate.

First, an aqueous solution of potassium persulfate (2.45 mM) was prepared, in which an appropriate amount of ABTS reagent was introduced to obtain a 7 mM solution of ABTS in an aqueous solution of potassium persulfate. The solution prepared in this way was incubated at 4 °C for 24 h and then diluted with methanol (50% v/v) to obtain an absorbance of approximately 1.000 ± 0.020.

The antioxidant activity of eugenol and its ester derivatives was measured as follows: 2500 µl of prepared ABTS solution and 25 µl of an ethanol solution with the tested antioxidant were introduced into the spectrophotometric cuvette. The cuvette was sealed with a stopper and then incubated for 6 min at room temperature. After this time, the spectrophotometric measurement was carried out at the appropriate wavelength and in triplicate.

This method is based on the use of the Folin–Ciocalteu reagent (takes place in an alkaline medium), which is used to determine the total content of phenolic compounds found in the tested samples. The reaction is based on the spectrophotometrically recorded color change of the test solution from yellow to blue.

Two hundred microliters of Folin–Ciocalteu reagent in 1800 µl of water was dissolved in a dark bottle. The solution prepared in this way was incubated at room temperature for 60 min. The antioxidant activity of eugenol and its ester derivatives was measured as follows: 1350 µl of distilled water and 1350 µl of sodium carbonate solution (0.01 mol/dm^3^) were introduced into the spectrophotometric cuvette with 150 µl of the prepared Folin–Ciocalteu solution and 150 µl of an ethanol solution containing the tested antioxidant. The cuvette was sealed with a stopper and then incubated for 15 min at room temperature. After this time, spectrophotometric measurements were carried out at the appropriate wavelength and in triplicate.

### Skin permeation studies of eugenol and new esters

Porcine skin was used for the study due to its similar properties permeability to human skin (Čuříková et al. 2017; Janus et al. 2020). The skin came from a local slaughterhouse. A fresh portion of skin from the abdomen was washed several times with a solution of PBS at pH 7.4. Skin with a thickness of 0.5 mm was cut with a dermatome, and then it was wrapped in aluminum foil and frozen at − 20 °C for a maximum of 3 months. This freezing time ensured the stability of the skin barrier properties (Badran et al. 2009). Before the examination, the skin was thawed at room temperature for about 30 min, and then it was soaked in a PBS solution for 15 min to hydrate it (Haq et al. 2018; Kuntsche et al. 2008; Simon et al. 2016). In the next stage, the skin was mounted in Franz diffusion cells.

The integrity of skin was checked 1 h after its installation in the Franz diffusion chamber (SES GmbH Analyze Systeme, Germany). Skin impedance was measured using an LCR 4080 meter (Conrad Electronic, Germany) operating in parallel mode at 120 Hz (kΩ error < 0.5%). To make the measurement, the tips of the probes were immersed in the donor and acceptor chambers filled with the PBS solution (Kopečná et al. 2017). Membranes with an electrical resistance of > 3 kΩ, corresponding to the resistance measured for human skin, were used in the study (Davies et al. 2004).

The penetration of eugenol and its ester derivatives was assessed in a Franz diffusion chamber consisting of a 2 ml donor chamber and an 8 ml acceptor chamber. The area through which the tested active ingredients permeated was 1 cm^2^. The acceptor fluid, mixed with a magnetic stirrer, was a PBS solution that maintained the physiological pH. The acceptor chamber was kept at a constant temperature of 37 ± 0.5 °C with the VEB MLW Prüfgeräte-Werk type 3280 thermostat. Before starting the test, Franz diffusion cells were allowed to equilibrate at 37 °C for 15 min. After this time, ethanol solutions of test compounds (at a concentration of 1% w/v) were placed in the donor chamber. For the study, 500 µL of the ethanol solution of the test compound (at a concentration of 1% w/v) was applied to the outer layer of the skin in each donor chamber to ensure continuous delivery of the active substance during the experiment. All donor chambers were closed with a plastic stopper to prevent excessive evaporation of the solution. The described tests were carried out for 24 h, while 0.3 ml of the solution located in the acceptor chamber was taken at specified intervals (30 min, 1 h, 2 h, 3 h, 4 h, 5 h, 8 h and 24 h), and then supplemented with a fresh portion buffer at the same pH (Kopečná et al. 2017). The samples were analyzed by high-performance liquid chromatography (HPLC) with a UV spectrophotometric detector (Knauer, Berlin, Germany). The components tested were separated on a 125 × 4 mm column containing Hyperisil ODS; particle size 5 µm. The flow rate of the mobile phase, consisted of acetonitrile, water, and MeOH (28:64:8, by vol) was 1 ml/min. Twenty microliters of each analyzed sample was injected onto the column.

After the experiment was carried out, the skin was extracted to estimate the residual volume of tested active ingredients accumulated in it. The antioxidant activity of the obtained extracts was also tested using modified methods described in (Brand-Williams et al. 1995; Nowak et al. 2017; Nowak et al. 2019). Extraction was carried out as follows: after the experiment was completed, the Franz diffusion chambers were dismantled, while the skin surface was washed three times with an aqueous solution of sodium lauryl sulfate (at a concentration of 0.5% w/w) to elute the ethanol solution of the test compound. A patch (1 cm^2^ diffusion surface) was cut from the skin prepared in this way, dried at room temperature, and then weighed and cut into smaller pieces. Then, 2 ml of concentrated methanol was added to it, and extraction was carried out for 24 h at 4 °C. After 24 h of incubation, the skin was homogenized (for 3 min) using a homogenizer (IKA®T18 digital ULTRA TURRAX, Germany). The extracts obtained were then centrifuged at 3500 rpm for 5 min. The supernatant was analyzed by HPLC to determine the content of active ingredients, while to evaluate the antioxidant activity of the obtained extracts the DPPH, Folin–Ciocalteau, and ABTS methods were applied.

The cumulative mass of active substance (µg) permeating into the receptor chamber was calculated based on the concentrations of compounds determined by HPLC. The permeation rate was determined based on the amount of permeation of a compound over a given period (µg/cm^2^/h). The accumulation of compounds in the skin was calculated by determination of remaining ingredients in the liquid obtained after skin extraction; the results are given in µg/cm^2^ of skin.

The antioxidant activity of solutions of test compounds applied to the skin, acceptor fluid taken after 24 h of penetration, and solutions obtained after skin extraction taken at the end of the experiment was determined using the DPPH, Folin–Ciocalteau, and ABTS methods, as described in sections: measurement of the antioxidant capacity of eugenol and new esters using the DPPH, the ABTS and the Folin–Ciocalteu methods.

Statistical calculations were done using Statistica 13 PL software (StatSoft, Polska). The results were evaluated using one-way analysis of variance (ANOVA). Significant differences between the permeation of individual compounds were evaluated using Tukey post-hoc test. Probabilities < 0.05 were considered to be statistically significant. Results are presented as the mean ± standard deviation (SD).

## Results

Research by the GC method showed that the process yielded eugenol esters as the main products with 100% selectivity, with eugenol conversion of 99.7% (in the case of EChA and ETChA) and 99.8% (in the case of EDChA).

**Fig. 3:**
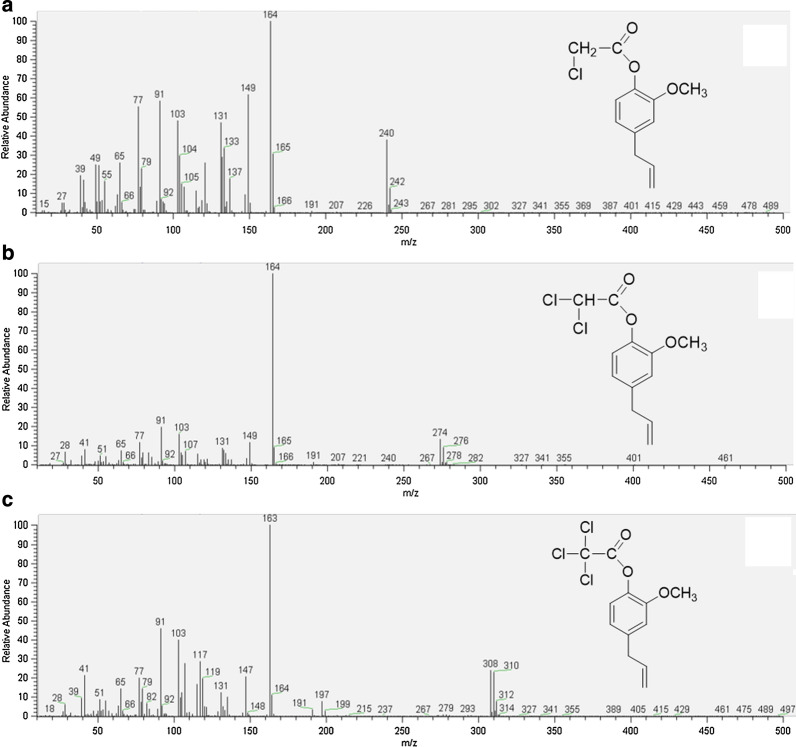
Mass spectra: **a** eugenyl chloroacetate (EChA), **b** eugenyl dichloroacetate (EDChA), and **c** eugenyl trichloroacetate (ETChA)

Based on the results obtained by the GC-MS method (presented in Fig. 3), in the esterification process of eugenol with chloroacetic acid chloride, one product was obtained with the following masses (m/z): 240 (73%), 242 (24%), and 243 (3%). These results correspond to the calculated mass of EChA (240.5 g/mol) (Fig. 3a). In the esterification process of eugenol with dichloroacetic acid chloride, one product was obtained with the following masses (m/z): 274 (56%), 276 (38%), and 278 (6%). The molecular weight of the identified product corresponds to the mass of EDChA (274.9 g/mol) (Fig. 3b). By esterifying eugenol with trichloroacetic acid chloride, one product was obtained with the following masses (m/z): 308 (43%), 310 (41%), 312 (15%), and 314 (1%). The molecular weight of the obtained ester corresponds to the mass of ETChA (309.5 g/mol) (Fig. 3c).

**Fig. 4:**
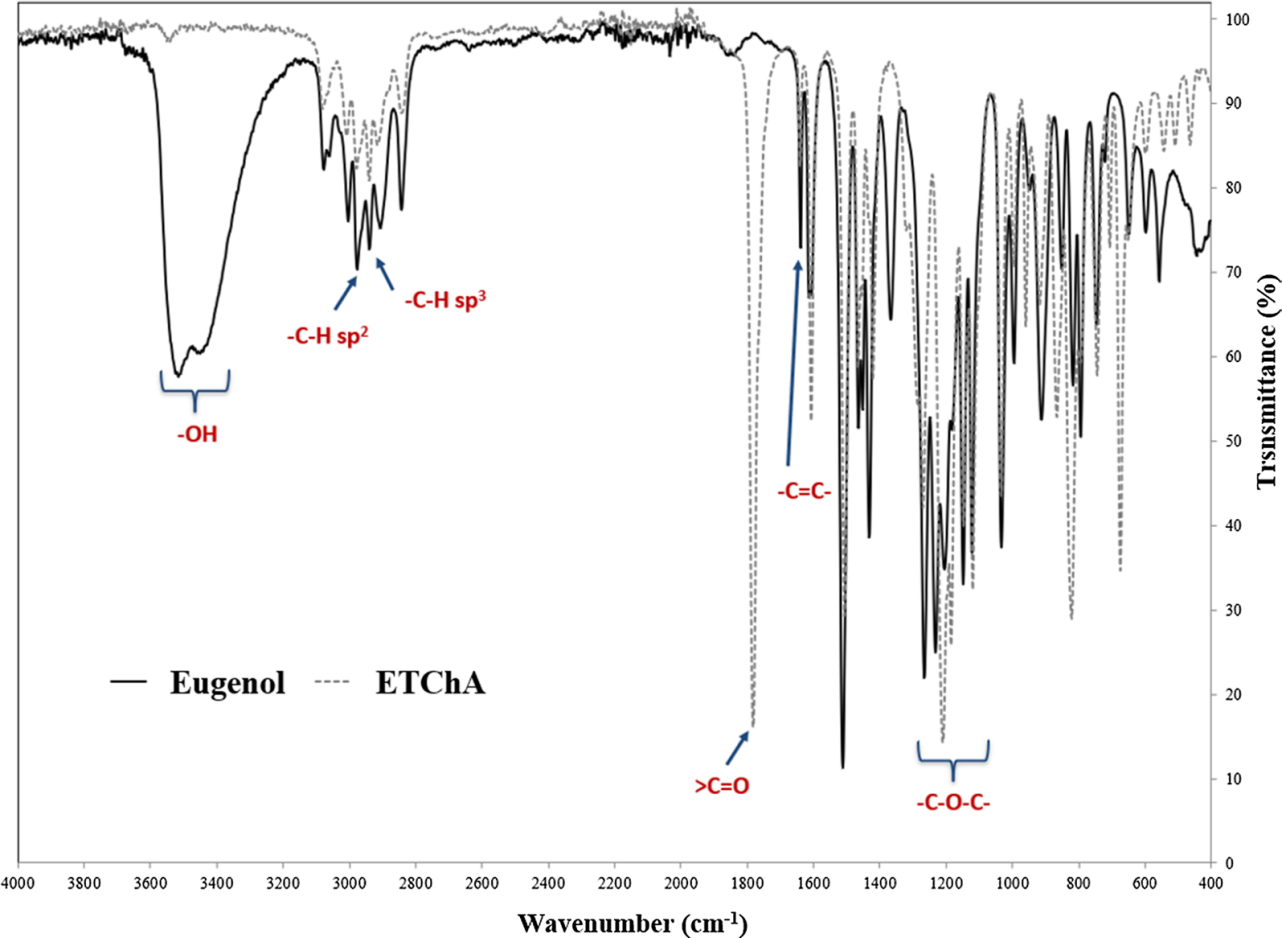
Example of the IR spectrum of eugenol and ETChA

The IR ETChA spectrum, which is shown in Fig. 4, has an absorption band characteristic of the ester group at a wavelength of about 1775 cm^−1^. Also, there is no wideband at a wavelength of about 3500 cm^−1^, which is attributed to the stretching vibration of O–H bonds. However, this band occurs in the eugenol molecule. Besides, absorption bands were observed at a wavelength of about 3010 cm^−1^ (derived from CH carbon atoms with sp^2^ hybridization) and at a wavelength of about 1637 cm^−1^ (associated with the presence of C= C carbon atoms), which occur in both the allyl group as well as in the aromatic ring of eugenol and its ester. In the IR spectrum of eugenol and ETChA, absorption bands were measured at wavelengths of about 2976 cm^−1^ (attributed to CH carbon atoms with sp^3^ hybridization) and about 1300–1118 cm^−1^ (found on carbon in the aromatic ring), derived from the methoxy group (Kuo et al. 2009; Ginting et al. 2019). In the case of the remaining esters (EChA and EDChA), the same functional groups that were present in the structure of ETChA were observed.

Table 1 presents a comparison of octanol/water partition coefficient values determined by the experimental method and calculated in the MestReNova program.

**Table 1:** Comparison of octanol/water partition coefficient values determined by the shake-flask method and calculated by the theoretical method

Compound	log P^1^ average value	log P^2^
Eugenol	2.20 ± 0.001c	2.49
EChA	2.43 ± 0.001ab	3.11
EDChA	2.65 ± 0.001ab	3.73
ETChA	2.75 ± 0.002a	4.35
DChAA	0.95 ± 0.002e	0.80
TChAA	1.51 ± 0.002d	1.42

^1^ determined by the shake-flask method; mean ± S.D. (n = 3)

^2^ log P calculated in the MestReNova program, different letters–values are significantly different, mass substance in the acceptor fluid (*P <* 0.001)

Each compound’s lipophilicity is represented as the decimal logarithm of the partition coefficient (Piwowarczyk et al. 2017). Results are presented as the mean ± standard deviation (SD). The partition coefficient (log P) values for eugenol and its ester derivatives, determined using the theoretical calculation method, were slightly higher than the log P values obtained by the spectrophotometric method (Table 1).

The log P values for dichloroacetic acid and trichloroacetic acid (DChAA 0.80 and 0.95 ± 0.002, TChAA 1.42 and 1.51 ± 0.002–—Table 1) were similar to the log P values described in the literature. However, by comparing the literature data describing the partition coefficient values determined by the experimental and theoretical methods, we found that depending on the method used, log P values ranged from 2.13 to 2.66 (in the case of eugenol), from 0.92 to 1.06 (in the case of dichloroacetic acid), and from 1.17 to 1.53 (in the case of trichloroacetic acid). There is no data in the literature describing the partition coefficient values that have been determined for ester derivatives such as eugenyl chloroacetate (EChA), eugenyl dichloroacetate (EDChA), and eugenyl trichloroacetate (ETChA). Eugenol ester derivatives are considered nonionized substances. Due to the neutral nature of these compounds, their solubility is not dependent on the pH of the environment. Under the conditions of the experiment, there is no dissociation of esters to ionized forms, which would significantly disturb the solubility profiles of the derivatives obtained (Kuo et al. 2009; Ginting et al. 2019; Pernin et al. 2019; Sangster 1993, 1997, 2009; Hansch 1996; Howard and Meylan 1997).

The values of the antioxidant activity of the tested compounds (at a concentration of 1% w/v) were 1.23 ± 0.01 mmol TE/dm^3^ (for eugenol), 0.32 ± 0.01 mmol TE/dm^3^ (for EChA), 1.08 ± 0.02 mmol TE/dm^3^ (for EDChA), and 1.04 ± 0.01 mmol TE/dm^3^ (for ETChA), respectively—Table 2. Both eugenol and its ester derivatives with the highest values of antioxidant activity (i.e., EDChA and ETChA) were assessed for penetration through pig skin using the Franz diffusion chamber.

**Table 2:** Antioxidant activity of 1% solutions of the test compounds applied to the skin, acceptor fluid obtained after 24 h of collection, and solutions obtained after skin extraction

Compound	Antioxidant activity (DPPH method)		
	Solution applied to the skin	Acceptor fluid after 24 h of penetration	Solution after skin extraction
	mmol TE/dm^3^		
Eugenol*	1.23 ± 0.01a	0.08 ± 0.01a	0.22 ± 0.02b
EDChA *	1.08 ± 0.02b	0.07 ± 0.00a	0.25 ± 0.01a
ETChA*	1.04 ± 0.01c	0.06 ± 0.00a	0.21 ± 0.01b
DChAA	No activity	No activity	No activity
TChAA	No activity	No activity	No activity

* Mean ± S.D. (n = 3), different letters - values are significantly different, mass substance in the acceptor fluid (*P <* 0.001)

Table 2 presents the results for the antioxidant activity of solutions of the tested compounds, carried out by the DPPH method.

Table 3 presents the results for the antioxidant activity of the solutions of the tested compounds, carried out using the ABTS method.

**Table 3:** Antioxidant activity of 1% solutions of the test compounds applied to the skin, acceptor fluid obtained after 24 h of collection, and solutions obtained after skin extraction

Compound	Antioxidant activity (ABTS method)		
	Solution applied to the skin	Acceptor fluid after 24 h of penetration	Solution after skin extraction
	mmol TE/dm^3^		
Eugenol*	4.74 ± 0.01a	0.25 ± 0.03a	0.91 ± 0.02c
EDChA *	4.70 ± 0.02a	0.19 ± 0.03a	1.14 ± 0.02a
ETChA*	4.71 ± 0.01a	0.20 ± 0.07a	1.03 ± 0.07b
DChAA	No activity	No activity	No activity
TChAA	No activity	No activity	No activity

* Mean ± S.D. (n = 3), different letters - values are significantly different, mass substance in the acceptor fluid (P < 0.001)

Table 4 shows the total polyphenol content in solutions of the tested compounds, carried out using the Folin–Ciocalteu method.

**Table 4:** Total polyphenol content in solutions of test compounds applied to the skin, acceptor fluid solutions obtained after 24 h of collection, and solutions obtained after skin extraction

Compound	Antioxidant activity (Folin–Ciocalteu method):		
	Solution applied to the skin	Acceptor fluid after 24 h of penetration	Solution after skin extraction
	mmol gallic acid/dm^3^		
Eugenol*	3.84 ± 0.05a	0.27 ± 0.01a	0.41 ± 0.03b
EDChA *	3.27 ± 0.10b	0.22 ± 0.01b	0.58 ± 0.04a
ETChA*	3.35 ± 0.06b	0.25 ± 0.01ab	0.55 ± 0.01a
DChAA	No activity	No activity	No activity
TChAA	No activity	No activity	No activity

* Mean ± S.D. (n = 3), different letters - values are significantly different, mass substance in the acceptor fluid (P < 0.001)

The test results, presented in Tables 2, 3 and 4, show that the initial solutions of the tested compounds (eugenol, EDChA, ETChA) applied to the skin, solutions of acceptor liquids, and solutions obtained after skin extraction were characterized by high antioxidant activity. In the case of studies carried out for pure TChAA and DChAA, no antioxidant activity was shown.

The results of the studies on the antioxidant activity of acceptor fluid solutions (collected after 24 h of permeation) showed that the tested solution were characterized by antioxidant activity due to of eugenol and its esters. The degree of reduction of the DPPH free radical increased in the following order: 0.08 ± 0.01 mmol TE/dm^3^ (in the case of eugenol) > 0.07 ± 0.00 mmol TE/dm^3^ (in the case of EDChA) > 0.06 ± 0.00 mmol TE/dm^3^ (for ETChA) (Table 2).

The antioxidant activity (determined by the ABTS method) of the compound solutions (eugenol, EDChA, ETChA) tested prior to application to the skin changed as follows: 4.74 ± 0.01 mmol TE/dm^3^ (for eugenol) > 4.71 ± 0.01 mmol TE/dm^3^ (for ETChA) > 4.70 ± 0.02 mmol TE/dm^3^ (for EDChA) (Table 3). The results of studies on the antioxidant activity of acceptor fluid solutions (collected after 24 h of permeation) showed that the eugenol solutions tested had the highest antioxidant activity (0.25 ± 0.03 mmol TE/dm^3^). Lower antioxidant activity was observed for the ETChA and EDChA solutions (0.19 ± 0.03 and 0.20 ± 0.07 mmol of TE/dm^3^) (Table 3).

Test results obtained by the Folin–Ciocalteu method showed that the polyphenol contents of the initial solutions applied to the skin changed as follows: 3.84 ± 0.05 mmol of gallic acid/dm^3^ (for eugenol) > 3.35 ± 0.06 mmol of gallic acid/dm^3^ (for ETChA) > 3.27 ± 0.10 mmol of gallic acid/dm^3^ (for EDChA) (Table 4). The results of tests for antioxidant activity of acceptor fluid solutions (collected after 24 h of permeation) showed that the tested eugenol solutions had the highest polyphenol content (0.27 ± 0.01 mmol gallic acid/dm^3^). Lower concentrations of 0.25 ± 0.01 and 0.22 ± 0.01 mmol of gallic acid/dm^3^, respectively, were measured for solutions of ETChA and EDChA (Table 4).

The results of studies on the antioxidant activity of solutions obtained after skin extraction taken after the experiment showed that both eugenol and its ester derivatives were characterized by high antioxidant activity, as estimated by the three techniques mentioned: DPPH, ABTS, and Folin–Ciocalteu. The degree of DPPH free radical reduction for these compounds increased in the following order: EDChA (0.25 ± 0.01 mmol TE/dm^3^) > Eugenol (0.22 ± 0.02 mmol TE/dm^3^ ) > ETChA (0.21 ± 0.01 mmol TE/dm^3^) (Table 2).

In contrast, the antioxidant activity (ABTS method) of the solutions obtained after skin extraction changed as follows: 1.14 ± 0.02 mmol TE/dm^3^ (for EDChA) > 1.03 ± 0.07 mmol TE/dm^3^ (for ETChA) > 0.91 ± 0.02 mmol of TE/dm^3^ (for eugenol) (Table 3). The research results obtained by the Folin–Ciocalteu method showed that the values of antioxidant activity of solutions obtained after skin extraction (ETChA and EDChA) were higher (0.55 ± 0.01 and 0.58 ± 0.04 mmol of gallic acid/dm^3^) than the values of antioxidant activity of eugenol (0.41 ± 0.03 mmol gallic acid/dm^3^) (Table 4).

Table 5 presents the results of studies on the penetration of active substances through pig skin and the amounts of extracted active ingredients accumulated in the skin.

**Table 5:** Results of studies on the penetration of active substances through pig skin and the amounts of extracted active ingredients accumulated in it

Compound	Mass of substance in the acceptor fluid after 24 h of penetration (µg)	Concentration of substance extracted from the skin (µg/cm^2^ skin)
Eugenol*	272.89 ± 54.64ab	566.93 ± 33.34a
EDChA*	302.77 ± 1.90b	555.09 ± 53.22a
ETChA*	297.62 ± 24.97b	539.14 ± 40.53a
DChAA*	247.63 ± 16.45ab	750.82 ± 44.62b
TChAA*	210.54 ± 16.45a	799.41 ± 39.99b

* Mean ± S.D. (n = 3**)**, different letters - values are significantly different, mass substance in the acceptor fluid (*P <* 0.05), concentration of substance extracted from the skin (*P <* 0.001)

**Fig. 5:**
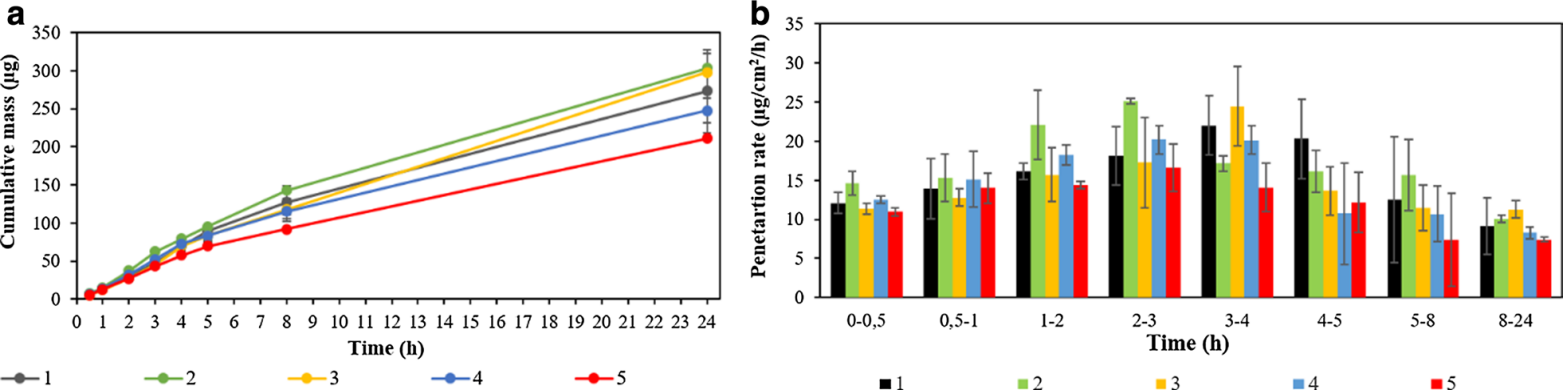
**a** Mass of test substance in the acceptor fluid during the 24-h experiment: (1) Eugenol, (2) EDChA, (3) ETChA, (4) DChAA, (5) TChAA. **b** The penetration rate of test substances through the skin during the 24-h experiment: (1) Eugenol, (2) EDChA, (3) ETChA, (4) DChAA, (5) TChAA

Figure 5 shows the results of studies on the permeation of active substances through pig skin during the 24-h experiment.

In our own research conducted in vitro, the penetration of eugenol and its new ester derivatives (EDChA, ETChA) through pig skin was assessed. For comparative purposes, tests were also carried out for pure acids (DChAA, TChAA). The experiment was carried out using a Franz diffusion chamber, in which the donor phase consisted of ethanol solutions of the compounds tested. The acceptor phase was PBS solution, because it corresponds to systemic conditions, is isotonic in nature, and allows conditions corresponding to the conditions prevailing in the deeper layers of the skin to be maintained (Alonso et al. 2014). Permeation of test substances through pig skin into the acceptor fluid increased in the following order: EDChA > ETChA > Eugenol > DChAA > TChAA. After conducting the experiment for 24 h, the highest average cumulative mass was observed in the case of EDChA (302.77 ± 1.90 µg). The mass was slightly lower in the case of ETChA (297.62 ± 24.97 µg) and for eugenol (272.89 ± 54.64 µg). In addition, the study showed that amount of ETChA penetration differed significantly from that of TChAA (210.54 ± 16.45 µg). The highest increase in the penetration rate of EDChA to the acceptor fluid (µg/cm^2^/h) was observed between 2 and 3 h, while for ETChA and eugenol, it was between 3 and 4 h (Fig. 5).

After the experiment was carried out, the skin was extracted in order to extract the tested active ingredients accumulated in it. The obtained test results showed that the concentration of tested substances in the analyzed extracts (µg/cm^2^ skin) decreased in the following order: TChAA (799.41 ± 39.99 µg/cm^2^ skin) > DChAA (750.82 ± 44.62 µg/cm^2^ skin) > eugenol (566.93 ± 33.34 µg/cm^2^ skin) > EDChA (555.09 ± 53.22 µg/cm^2^ skin) > ETChA (539.14 ± 40.53 µg/cm^2^ skin). Pure TChAA and DChAA acids were accumulated in the skin, as evidenced by the high concentrations of compounds found in the analyzed extracts: TChAA (799.41 ± 39.99 µg/cm^2^ skin) and DChAA (750.82 ± 44.62 µg/cm^2^ skin) (Table 5). The factor significantly affecting the transport of active substances is the lipophilicity of the test compound (Yang et al. 2008; Srinivas et al. 2019; Ichihashi et al. 2003; Casagrande et al. 2007). The optimal log P coefficient (which is an indicator of the lipophilicity of the active substance) is in the range of 2 to 3 (Janus et al. 2020; Haq et al. 2018; Simon et al. 2016). This was also confirmed in our own research, which showed that both eugenol and its ester derivatives were characterized by good permeability through the skin (log P: Eugenol 2.20 ± 0.001, EDChA 2.65 ± 0.001, ETChA 2.75 ± 0.002, Table 1). Lipophilic compounds penetrate much more easily through the skin, because the skin consist mainly of lipid substances which, at the same time, limits the penetration of hydrophilic substances (log P: DChAA 0.95 ± 0.002, TChAA 1.51 ± 0.002, Table 1) commonly used in dermatology or cosmetology (Jaworska et al. 2011). The lower lipophilicity of the compound (log P < 2) is associated with its worse penetration the skin (Simon et al. 2016). This relationship was also confirmed in our own research; the average masses accumulated in the acceptor fluid after 24 h were 247.63 ± 16.45 (in the case of DChAA) and 210.54 ± 16.45 (in the case of TChAA), as shown in Table 5, while the average cumulative masses for these compounds at 0.5; 1; 2; 3; 4; 5; 8, and 24 h are shown in Fig. 5.

## Discussion

The antioxidant activity of eugenol and EDChA depends on the concentration of antioxidant in the test sample. As it has been shown in our previous studiy (Makuch et al. 2019), the antioxidant activity of the sample increased as from 76% (for 0.25 µg/ml) to 88% RSA (for 5 µg/ml). The antioxidant activity of eugenol increases with an increase of this compound concentration in the tested sample due to a reduced electron density on the oxygen atom of the phenolic group (i.e. the –OH group associated with carbon of the aromatic ring). The hydrogen bonding energy is much lower, which makes it easier to give it to the DPPH radical (through the reaction of the radical and antioxidant molecule with the formation of the adduct). Other studies have shown that the values of the parameter determining the concentration reducing 50% of free radicals (IC_50_) for eugenol are inversely proportional to its antioxidant activity. The lower the IC_50_, the higher the antioxidant activity (Makuch et al. 2019).

The results of EDChA antioxidant activity presented in Table 2, showed that the antioxidant activity may be strongly influenced by the kinetic behavior of this compound. As our previous research has shown (Makuch et al. 2019), by increasing the concentration of EDChA in the tested sample, a decrease in antioxidant activity was observed: from 93% (at 0.25 µg/ml ester concentration) to 89% RSA (at 5 µg/ml ester concentration). The reason for under-activation is that the reaction time (10 min) between DPPH and EDChA is too short. As a result, this time prevents the end of the reaction between radical and antioxidant. This phenomenon is most often observed in the case of antioxidants (essential oils, compounds isolated from plant materials or plant extracts) characterized by a slow reaction with DPPH radical (Cenobio-Galindo et al. 2019; Fadda et al. 2014; Magalhaes et al. 2012; Ahmad et al. 2012).

Eugenol has a hydroxyl group (–OH) associated with an aromatic ring with acidic properties, which could lead to antioxidant activity. Its free radicals scavenging activity could lead to form phenolic radicals. These radicals are stable due to resonance caused charge transfer and are not able to detach hydrogen from lipid or protein molecules (and to decrease the oxidation).

Replacement of hydrogen atoms in the aliphatic chain EChA, EDChA and ETChA by heteroatoms (in this case, chlorine atoms) enhances the antioxidative properties. Eugenol esters containing chlorine atoms in the structure easily trap free radicals, giving up the H atom in the aliphatic chain. The reason is a change in the shape of the molecule, i.e. a change in length, direction, range and polarization of the bonds and a change in the symmetry of the particles. Introduction of chlorine atoms into the structure, causes polarization of bonds between carbon-chlorine atoms. The polarization of bonds between the carbon-chlorine atoms reduces the density of the electron cloud in the whole molecule and causes polarization of all close bonds present in the structure. As a result of this bond between the carbon-hydrogen atoms in EChA, EDChA and ETChA molecules, they change their length and polarity. In addition, the presence of chlorine atoms in the structure of these compounds changes the electro-neutrality of carbon atoms. Additionally, the presence of the methoxyl group (–OCH_3_) in the eugenol molecule and the EChA, EDChA and ETChA molecules increases the antioxidant properties of these compounds (Rice-Evans et al. 1996).

We demonstrated that new eugenol derivatives (EDChA and ETChA) containing ester groups, penetrate more readily through biological membranes. They are characterised by higher partition coefficient compared to the parent output eugenol and acids (DChAA and TChAA), which can have a positive impact on enhance active substance transport through biological membranes. Eugenol is a terpene compound classified as an absorption promoter that is characterized by high antibacterial (Makuch et al. 2016; Pavithra 2014) as well as antioxidant activity (Hamed et al. 2012; Promod et al. 2010. Terpenes, which are a group of substances that are commonly considered safe from the point of view of dermal toxicity, are often used in preparations applied to the outer layer of the skin (Makuch et al. 2017; Ahad et al. 2016; Pavithra 2014).

The transport of the active substance the skin also depends on the molecular weight of the active substance itself. Overcoming the lipophilic barrier, which is the skin, is possible for non-polar—new esters of eugenol, which have molecular weights of < 600 Da (Malinowska et al. 2013; Tan et al. 2015; Thi and Hwang 2014). Besides better absorption of active substance by penetrating faster, the presented esters can provide endogenous action against free radicals, which is their superiority to the used in cosmetology acids (α- and β-hydroxy acids, TChAA), for which the skin is a barrier limiting their penetration. Data in the literature indicate that after a single application of trichloroacetic acid, complete epidermal necrolysis occurs, resulting in a decrease in capillary permeability. Therefore, after application of TChAA, no irritation or inflammation of the skin was observed (Sarkar 2012; Pathan and Setty 2009). In contrast, these symptoms occur after the application of commonly used α- and β-hydroxy acids (AHA and BHA). EDChA and ETChA are alternatives to AHA, BHA, and TChAA, which are active substances commonly used in problem skin care that have a tendency to cause imperfections (Karolak et al. 2017; Pathan and Setty 2009).

In addition, the good permeability of EDChA and ETChA through the skin and their proper accumulation in the skin (Table 5; Fig. 5) as well as their antioxidant capacity (Tables 2, 3 and 4) can limit also the exogenous effects of free radicals. Free radicals are highly reactive, toxic molecules due to the presence of one or more impaired electrons. Within tissue they damage DNA, proteins, lipids and carbohydrates (Dhale et al. 2007). The reactivity of new esters of eugenol by donating their electrons to the free radicals suggested use EDChA and ETChA as antioxidant.

In recent years, antioxidants have increasingly been used as active ingredients in cosmetic and pharmaceutical preparations, because they act as ROS blockers. Among the huge group of antioxidants, clove oil and eugenol (the main component of clove oil) can be distinguished as having confirmed antioxidant and antimicrobial effects. Although eugenol, as a biologically active substance, is already used in many cosmetic and pharmaceutical preparations the antioxidant effect of eugenol after crossing the skin barrier has not previously been studied. In addition, the use of new eugenol derivatives (EDChA, ETChA) as biologically active substances increases their penetration (as confirmed in our own research) of the skin and could decrease the toxic effect of exogenous and endogenous ROS. The obtained derivatives were characterized by high antioxidant activity, which was estimated after 24 h of conducting the experiment, which indicates long-term protection against ROS in the deeper layers of the skin. In the presented studies, the relationship between the lipophilicity of each tested active substance and its antioxidant capacity was estimated. The research presented in this publication thus provides new knowledge to this area of research (Li et al. 2011; Makuch et al. 2016; Makuch et al. 2015; Makuch and Kądziołka 2018; Wróblewska et al. 2017; Makuch et al. 2017; Makuch and Wróblewska 2014; Pavithra 2014; Hamed et al. 2012).

## Data Availability

Not applicable.
